# Adaptive Graph Learning with Persona-Aware Attribution for Event-Resilient Metro Passenger Flow Prediction

**DOI:** 10.3390/s26196316

**Published:** 2026-10-06

**Authors:** Ahmed Raza, Guangjie Liu, Jie Cheng, Weiqian Wu, Muhammad Usman Shoukat

**Affiliations:** 1 School of Electronic and Information Engineering, Nanjing University of Information Science and Technology, Nanjing 210044, China; ahmedraza@nuist.edu.cn (A.R.); 202212490705@nuist.edu.cn (J.C.); 202412180618@nuist.edu.cn (W.W.); 2 Energy and Transportation Domain, Beijing Institute of Technology, Zhuhai 519088, China; usmanryk12@gmail.com

**Keywords:** passenger flow prediction, graph attention networks, smart card data, spatiotemporal signal processing, sensor networks, intelligent transportation systems

## Abstract

**Highlights:**

**What are the main findings?**

- Adaptive graph learning reduces RMSE by 8.56% versus static topology.
- TDAG-Net achieves best event-day RMSE 32.31 across 191 stations.
- Persona graphs are 98.55% identical; splitting inputs degrades RMSE 10.36%.
- Tourist share triples from 5.7% to 16.5% during network-wide event peaks.

**What are the implications of the main findings?**

- Metro event management should prioritize unfamiliar visitor riders.
- Adaptive adjacency should replace static POI graphs in forecasting.
- Ticket–persona decomposition aids interpretation, not model input.

**Abstract:**

Urban rail passenger flow forecasting, based on large-scale automatic fare collection (AFC) sensor networks, is typically evaluated on ordinary days, yet operators most need accurate predictions during event days. The AFC infrastructure comprises 191 sensor-equipped stations with turnstile transducers that generate 95.7 million discrete sensing events (inboard/outboard readings) over 57 days, constituting a high-velocity, multimodal spatiotemporal sensor stream. This study presents a topology-driven adaptive graph network (TDAG-Net) for event-day forecasting across a full metro system. The model learns spatial dependencies end-to-end by merging multi-scale temporal convolution with an adaptive graph attention branch through learned gates. An attention long short-term memory (LSTM) then decodes 60-min forecasts for all 191 stations at once. A ticket–persona module reads 25 fare channels as four rider types to decompose demand surges by traveler profile. The proposed model reduces root mean square error (RMSE) by 9.29% and achieves the best performance on 22 flagged event days. Ablation reveals that adaptive adjacency is the dominant component and reduces horizon decay to 17.3% from 33.0%. Counter to expectation, persona-specific graphs are 98.55% identical, and splitting inputs by rider type degrades accuracy by 10.36% RMSE. On event peaks, the tourist/visitor share rises from 5.7% to 16.5%, indicating that event-day crowd management should prioritize unfamiliar riders over regular commuters.

## 1. Introduction

Urban rail transit is now the backbone of sustainable mobility in large cities, with measurable impacts on quality of life and urban development [1]. The world’s first subway opened in London in 1863 [2]. Since then, these systems have become vital parts of modern urban infrastructure. They now carry millions of passengers every day. Short-term passenger flow prediction supports real-time crowd management, train scheduling, and passenger information systems [3], while complementary sensing technologies such as camera-based vehicle speed estimation further enhance network-level situational awareness [4]. The stakes are highest exactly when prediction is hardest: during concerts, sporting fixtures, and public holidays demand surges deviate sharply from habitual commuting rhythms and the operational cost of a missed peak is measured in overcrowded platforms and safety risk [5].

Methodologically, the literature has evolved through three stages: classical statistical models, recurrent and convolutional sequence models, and spatial–temporal graph neural networks that directly encode metro topology. Classical forecasters established the problem’s statistical structure. SARIMA captured railway seasonality [6]; ARIMA with wavelet decomposition handled non-stationarity [7]; and Kalman filtering combined with k-nearest neighbors delivered robust short-term estimates [8]. Deep sequence models later became widely used. LSTM [9] and GRU variants [10,11] became common choices, with improvements for long-term patterns [12], origin–destination forecasting [13], and cluster-based training [14]. Temporal convolution offered a parallelizable alternative [15], hybridized with LSTM in 1DCNN-LSTM [16] and Conv-LSTM congestion-delay models [17], while Chen et al. [18] extended these ideas to scale-adaptive forecasting with multiscale horizons and coupled graph interaction. Fully connected and autonomous spatiotemporal stacks [19,20], ST-LSTM [21], and Gaussian-process or kernel-ridge regressors [22] rounded out the pre-graph era. These models capture temporal regularities well but are blind to network topology.

Graph signal processing [23] supplied the mathematical foundation for learning on irregular domains, and recent work such as DADiffNet has advanced adaptive subgraph learning for large-scale traffic forecasting [24]. Metro-specific variants proliferated: T-GCN [25], graph CNN-LSTM hybrids [26], incident-aware GCRNNs [27], spatiotemporal multi-graph convolution for ride-hailing [28], GCN-ResNet for urban rail [29], temporal multi-graph networks for multi-station prediction [30], dynamic hypergraph convolution for metro flow [31], multi-graph convolutional-recurrent networks [32], and TMFO-AGGRU [33]. Auxiliary context has also been used, including multi-source data fusion [34], smart-card-based deep prediction [35], and special-event modelling using smart-card data. Two observations motivate our design: nearly all of these models treat the passenger mass as homogeneous, and even adaptive-graph models are rarely stress-tested on flagged event days at network scale. Recent event-conditioned studies have explicitly modeled special-event and holiday effects, including multimodal deep learning for subway flow under events [36], GCN-Transformer forecasting for holidays [37], and dynamic multigraph spatiotemporal graph convolution for urban rail flow [38]. Smart sensor-based card research has also moved beyond aggregate prediction toward passenger profiling and behavioral inference [39,40,41,42,43]. These studies motivate our distinction between aggregate forecasting and forward-looking passenger-profile attribution.

Our own research work has followed this arc: TGALSTM fused a temporal convolutional network, a graph attention network over static POI similarity, and an attention-LSTM for multi-step forecasting [44]; a companion framework integrated multi-factor contextual signals through a CNN-BILSTM-Attention approach pipeline [45]; and canonical graph forecasters, such as DCRNN [46], STGCN [47], Graph WaveNet [48], and AGCRN [49], now define the accuracy frontier for network-wide prediction, with recent work further improving performance through dimensional balance in large-scale spatiotemporal models [50].

Yet four gaps remain open. First, passengers are modelled as one homogeneous mass: a commuter and a tourist are indistinguishable in the aggregate scalar, although their behavior diverges most violently during events. Second, the spatial graph is fixed beforehand, so TGALSTM’s POI-similarity adjacency [44] never adapts to the functional connectivity that ridership co-variation reveals. Third, evaluation remains narrow: the predecessor was validated on 78 stations across 60 working days with a 9:1 train/test split, but event days were never isolated for analysis and no modern GNN baselines were compared [3]. Fourth, no existing forecaster can say which kind of passenger drives a predicted surge, an interpretability gap that matters when operations staff must decide where to deploy crowd control.

This work began with the opposite hypothesis. We hypothesized that conditioning heterogeneous graph branches on ticket-derived personas would sharpen both accuracy and explanation. The data foundation for this study is the Nanjing Metro AFC sensor network. Each station is equipped with turnstile gates fitted with contactless smart-card readers, QR-code scanners, and token validators that act as distributed sensor nodes. Every passenger tap generates a discrete sensor event comprising station ID, timestamp, ticket channel, and direction (entry/exit). The raw sensor stream is inherently noisy, particularly subject to missed taps, duplicated records, corrupted timestamps, and anomalous station IDs, and exhibits severe distribution shifts during large public events. This paper treats passenger flow prediction as a sensor-data perception task: learning to forecast future graph states from historical sensor observations. The data clearly showed that this approach did not improve the model. Persona-specific graphs learned from the 2023 Nanjing AFC corpus turned out 98.55% identical (mean pairwise cosine similarity 0.9855), and splitting flow into persona channels degraded RMSE from 31.95 to 35.26, because the Tourist/Visitor (5.7% share) and Concession/Student (8.2%) channels lack the signal-to-noise ratio for independent spatial branches. The breakthrough lay elsewhere: a unified-flow architecture with end-to-end learned adaptive adjacency. Therefore, we use a clearer approach: persona decomposition supports interpretation, while adaptive graph learning drives prediction.

The resulting model, TDAG-Net, is validated across the Nanjing Metro using 95.7 million entry AFC records (190.9 million entry and exit events; median 3.30 million events/day) from 191 stations on 13 lines over 57 days and 819 test windows. This expands coverage from 78 to 191 stations, a 2.45× increase. It reduces RMSE by 9.29% over TGALSTM (31.95 vs. 35.22), attains the lowest event-peak error of all nine baseline models (EPE 16.08), ranks first on the 22 flagged event days (RMSE 32.31, beating AGCRN by 4.97% and TGALSTM by 9.37%), and degrades only 17.3% from step 1 to step 6 against TGALSTM’s 33.0%, while remaining within 3.5% of AGCRN overall.

The contributions are threefold:

- Adaptive graph learning: Replaces static POI connections with data-learned station links, giving the largest improvement (+8.56% RMSE when removed).
- Ticket–persona decomposition: Groups 25 ticket channels into four personas and provides quantitative explanations for predicted or observed peaks.
- Network-wide evaluation: Tests the TGALSTM-based model across 191 stations and 819 test windows, including 182 event windows, against nine baseline algorithms and matched architectural ablations.

Section 2 describes the dataset and formalizes the task. Section 3 presents the seven-stage architecture. Section 4 reports overall, event-day, horizon, and topology-sensitivity results. Section 5 analyzes interpretability. Section 6 discusses positioning, limitations, and deployment, and Section 7 concludes.

## 2. Dataset and Problem Formulation

### 2.1. Nanjing Metro AFC Corpus

**Figure 1:**
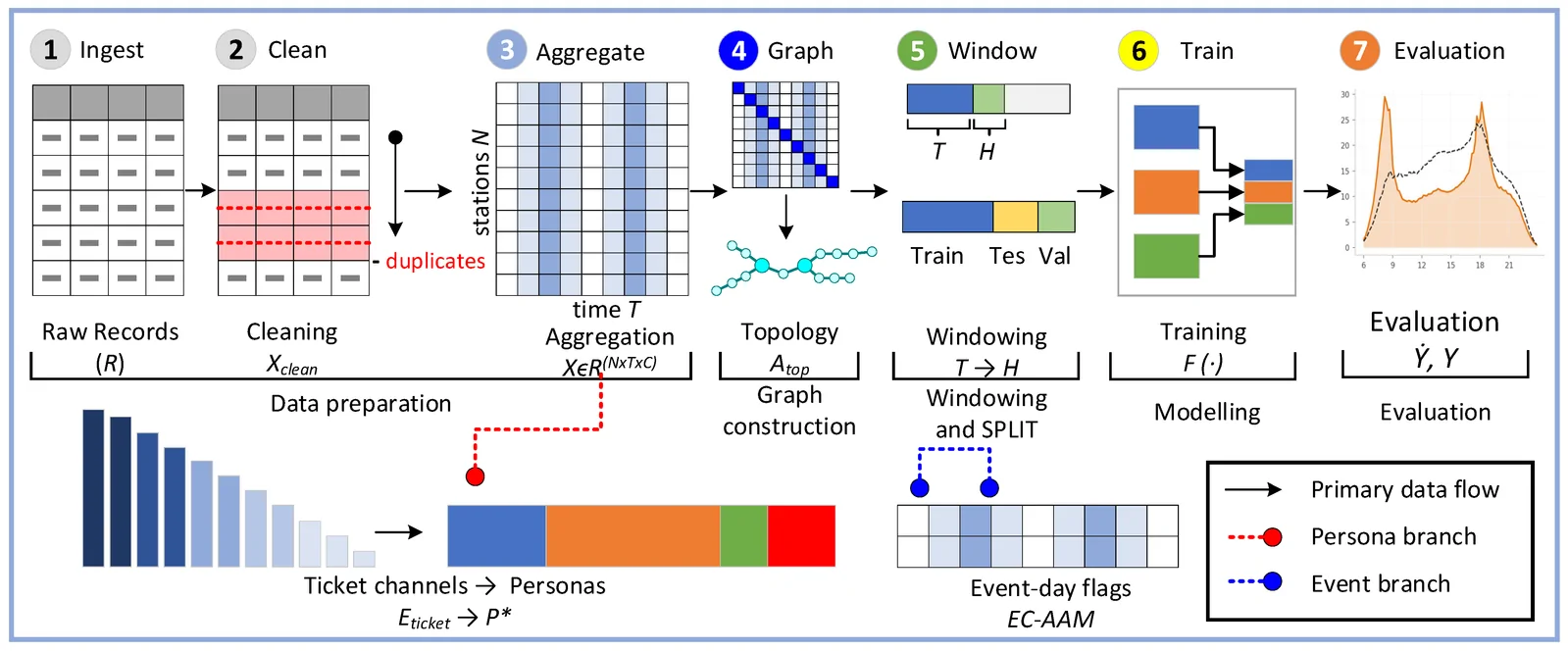
End-to-end data sensor–data processing pipeline: ingestion of 95.7M clean AFC sensor readings (turnstile tap events), records cleaning, 10-min aggregation, correlation-based graph construction, windowing and splitting, TDAG-Net training with the TD-PDM side channel, the extraction of Personas (P^*^) from ticket channels (*E_ticket_*) and event-conditioned evaluation.

Our primary corpus is the complete AFC transaction log of the Nanjing Metro for 1 March to 31 May 2023. Figure 1 summarizes the end-to-end data pipeline. The AFC system functions as a distributed sensor network where each turnstile acts as a sensing node. After deduplication of 8.65 million sensor readings and repair of corrupted epoch timestamps, 95,733,909 clean entry-direction sensor events were retained and aggregated into 10-min station-level intervals across the 06:00 to 23:50 service window (108 steps per day), yielding 57 usable days. These entry records correspond to 190.9 million total system-wide AFC tap events (combining entry and exit transactions), reflecting a median daily ridership of 3.30 million total AFC tap events (mean 3.35M events/day). The asterisk (∗) indicates that these are derived latent representations, specifically the inferred per-persona flow contributions, rather than raw observations. Table 1 lists the corpus properties.

**Figure 2:**
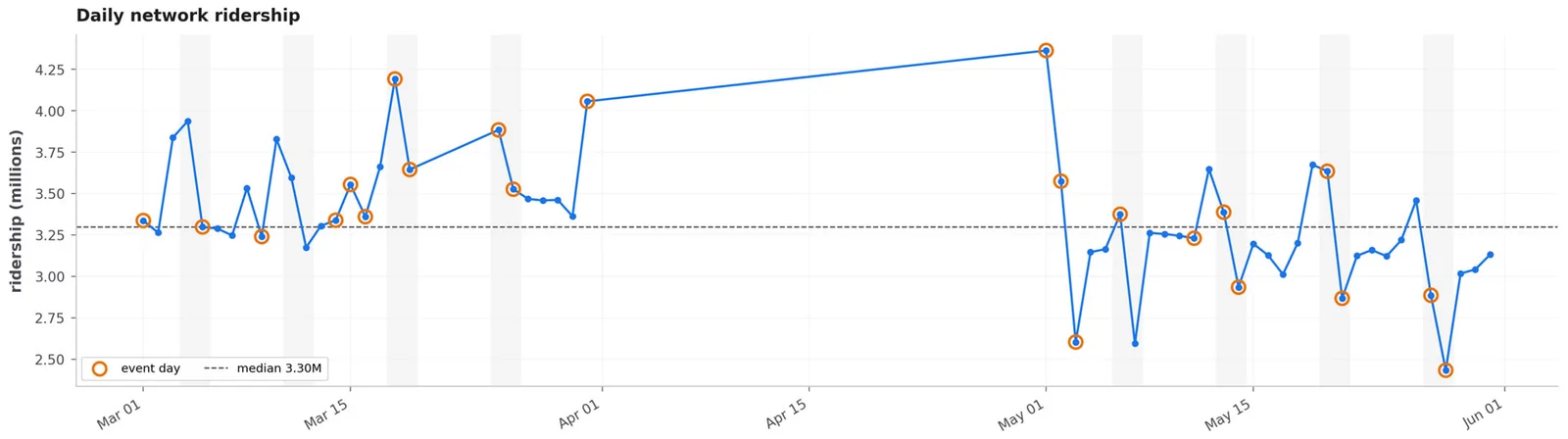
Daily ridership of the 2023 Nanjing Metro across the 57-day study window (median 3.30M daily entry+exit AFC events, totaling 190.9M events across 57 days). Weekend bands are shaded; the 22 automatically flagged event days are circled.

Figure 2 plots the resulting daily ridership across the 57-day study window, punctuated by 22 flagged event days. Three distinct graph objects are used throughout the study: the physical topology matrix *A_topo_* ∈ ℝ*^N^*^×*N*^, derived from rail-track connectivity and interchange stations, representing the fixed physical infrastructure of the metro network; the correlation topology *A_corr_* ∈ ℝ*^N^*^×*N*^, obtained from Pearson flow correlations and specified with threshold *ρ* = 0.30 and top-k = 8; and the learned adaptive adjacency *A_adapt_* ∈ ℝ*^N^*^×*N*^, initialized from *A_corr_* but optimized end-to-end. Thus, correlation topology is used as a data-derived prior, not as a substitute for the physical network topology.

### 2.2. Ticket Channels and Passenger Personas

**Figure 3:**
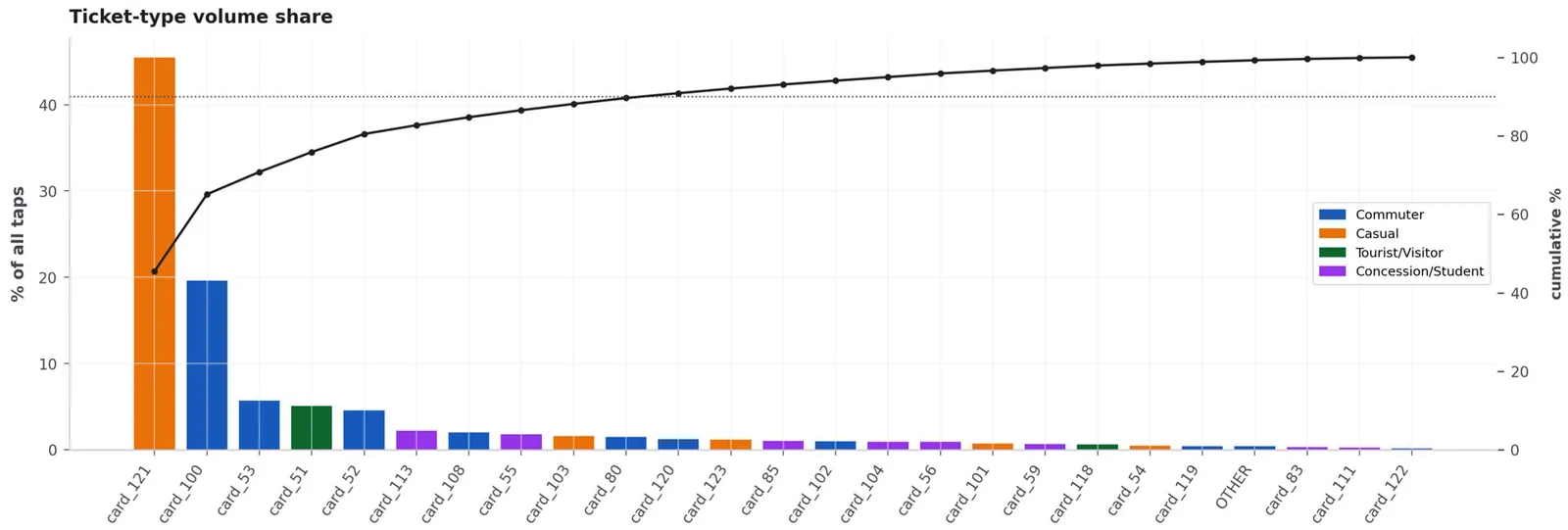
Ticket-channel Pareto. The 25 active channels cover 99.58% of taps; card_121 (≈45.5%) and card_100 (≈19.6%) dominate. TD-PDM’s soft assignment maps every channel to one of four personas.

The AFC system distinguishes 25 active ticket channels covering 99.58% of all taps. The channel distribution is highly uneven (Figure 3): card_121 accounts for about 45.5% of taps and card_100 for 19.6%, followed by a long tail of QR, token, and concession media. TD-PDM maps these channels into four passenger personas: Commuter (36.6%), Casual (49.5%), Tourist/Visitor (5.7%), and Concession/Student (8.2%). The mapping is not based on fare-media labels alone. It is grounded in 11 behavioral signature features: weekend-to-weekday demand ratio, AM peak share, PM peak share, midday share, late-night share, peak concentration, AM/PM asymmetry, spatial entropy, Herfindahl–Hirschman Index (HHI), entry/exit directional balance, and daily burstiness. Volume-weighted k-means clustering followed by Hungarian assignment was used to form the four behavioral groups. The interpretation was additionally cross-checked against known Nanjing fare-product rules and registration constraints, so the persona names are treated as empirical behavioral labels rather than direct claims about individual identity or travel purpose.

**Figure 4:**
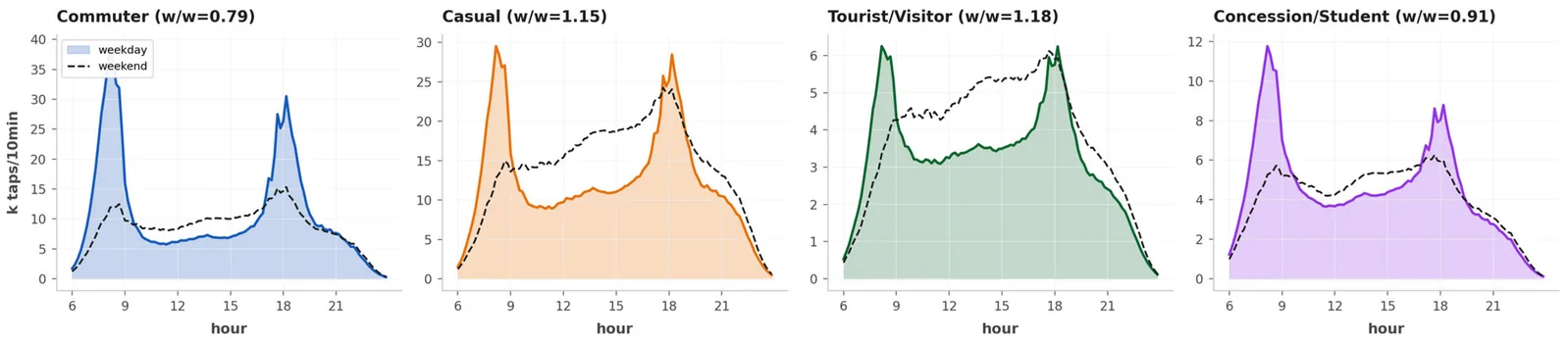
Persona behavioral profiles. Weekend-to-weekday ratios (Commuter 0.79, Casual 1.15, Tourist/Visitor 1.18, Concession/Student 0.91) show distinct travel patterns that aggregate models may miss.

Their travel patterns differ clearly (Figure 4): weekend-to-weekday demand ratios are 0.79, 1.15, 1.18, and 0.91, respectively. Commuter channels correspond primarily to registered stored-value or monthly-pass products and show stronger AM/PM regularity; Casual channels are dominated by single-fare QR/token use and more distributed travel; Tourist/Visitor channels are associated with intercity or out-of-town products and higher weekend and spatial diversity; and Concession/Student channels correspond to student and senior concession products with characteristic midday/afternoon patterns. These mappings are used only to support group-level interpretation. Raw station tap logs at event venues also confirm the observed increase in out-of-town and visitor-oriented fare-media transactions during event hours, supporting the reported increase in the Tourist/Visitor share.

### 2.3. Problem Formulation

The chronological split contains 40 training days (1 March to 14 May), 8 validation days (15–22 May), and 9 test days (23–31 May). With 108 service bins per day, L = 12 and H = 6 yield 108 − 12 − 6 + 1 = 91 valid windows per complete day, giving 3640 training, 728 validation, and 819 test windows. Windows are generated strictly within each calendar day and never cross the overnight service closure or a split boundary. Of the 22 corpus-wide event days, exactly two occur in the test split (27–28 May), producing 2 × 91 = 182 event test windows.

Event-day construction was performed offline for benchmark curation using a robust day-of-week surge detector. Let *m_d_*_,*w*_ and *MAD_d_*_,*w*_ denote the median and median absolute deviation of daily peak 10-min entry flow for day-of-week *w*. A station-day candidate is flagged when the robust Z-score is at least 4.0 and the observed peak is at least 1.5× the corresponding day-of-week median. This procedure identifies 155 localized station-surge candidates across the 57-day corpus. A calendar day is included in the 22-day event benchmark when at least one station exhibits an extreme surge or the day coincides with an official public holiday. For forecasting, however, the exogenous event flag and countdown are taken strictly from a priori public event calendars and scheduled fixtures; the observed-flow detector is not used to generate the model input, preventing future-information leakage.

Let the metro network be represented as a graph *G* = (*V*,*E*) with *N* = |*V*| = 191 stations. At each 10-min time step *t*, the network state is captured by the flow tensor *X_t_* ∈ ℝ*^N^*^×*C*^, where *C* = 1 denotes the single input channel corresponding to total tap-in counts, together with exogenous context *z_t_* (time-of-week encoding, day-of-week, event flag, and transfer ratio). Given a lookback window of *T* = 12 steps (120 min), the task is to learn a forecasting function *F*(⋅) that predicts the next *H* = 6 steps (60 min) for all *N* = 191 stations simultaneously, formalized as,

(1) $$\hat{Y}=F(X,z;A_{\mathrm{adapt}},A_{\mathrm{topo}})$$

where *X* ∈ ℝ*^N^*^×*T*×*C*^ is the input tensor of historical flows, *z* is the sequence of exogenous context vectors, *A_adapt_* ∈ ℝ*^N^*^×*N*^ is the fixed physical-topology adjacency matrix derived from rail-track connectivity and interchange stations, and *A_topo_* is a learned adaptive adjacency matrix optimized end-to-end to capture latent data-driven station dependencies. *A_corr_* is used only to initialize the adaptive embeddings through the correlation-based prior. The output *Y* contains the forecasted passenger flows for all stations over the prediction horizon.

## 3. TDAG-Net Architecture

### 3.1. Overview

**Figure 5:**
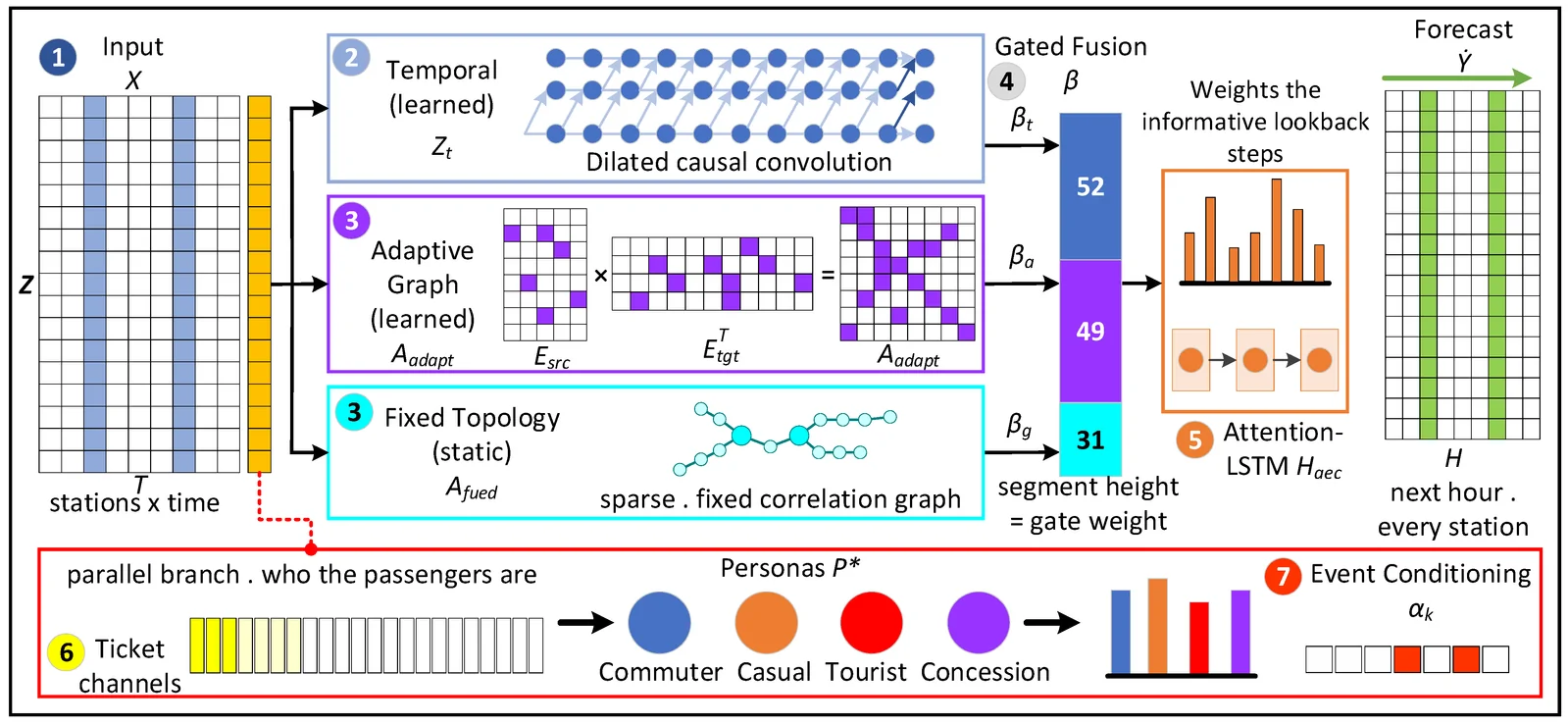
TDAG-Net architecture. The unified-flow engine (ST-TCN, AGA-Net, gated fusion, attention-LSTM) drives prediction.

Figure 5 shows the seven-stage pipeline. (1) The unified aggregate flow tensor and exogenous context enter (2) a multi-scale temporal convolutional encoder and (3) an adaptive graph attention network whose adjacency is learned; (4) three learned gates fuse temporal, adaptive-spatial, and topology-fixed features; (5) an attention-LSTM decodes the fused encoding into *H* = 6 forecasts for all 191 stations. In parallel, (6) the TD-PDM branch decomposes flow by ticket persona, and (7) event-conditional analysis modulates persona contributions during flagged windows. P^*^ denotes the derived set of persona-specific flow representations (Commuter, Casual, Tourist, Concession) extracted from raw ticket channels. TD-PDM branch provides per-persona interpretability with learned event-volatility weights *α_k_*. Fusion gates stabilized at *β* = 0.388/0.366/0.246 (temporal/adaptive/topology).

### 3.2. Multi-Scale ST-TCN

The temporal encoder applies dilated causal convolutions with dilations [1,2,4,8] across 64 channels, capturing patterns from 10-min micro-cycles to 80-min commuting waves. A local sigmoid gate and channel-attention mechanism, inherited from the enhanced TCN in TGALSTM [44], are used to refine the extracted features. The sigmoid gate controls the contribution of each local feature by assigning higher weights to more important information and lower weights to less relevant information. Channel attention then evaluates the importance of different feature channels and gives greater weight to channels that contain useful patterns. Operating on unified flow rather than persona channels preserves the full signal-to-noise ratio at every station.

### 3.3. Adaptive Graph Attention

This is the primary novel component. Following the self-adaptive paradigm of Graph WaveNet and AGCRN, we learn an adjacency matrix directly from data rather than relying solely on a fixed physical topology. The adaptive adjacency matrix is defined as,

(2) $$A_{\mathrm{adapt}}=\mathrm{Softmax}\left(\mathrm{ReLU}(E_{\mathrm{src}}\cdot E_{\mathrm{tgt}}^{⊺})\right),\;E_{\mathrm{src}},E_{\mathrm{tgt}}\in R^{N\times 16}$$

The source and target embeddings are learnable 16-dimensional vectors. Their initial values are obtained from a truncated SVD of the correlation topology *A_corr_*, after which the embeddings remain fully learnable during training. This initialization supplies a data-derived starting point while allowing the model to discover functional station dependencies that differ from both physical connectivity and the initial correlation graph. A four-head graph attention layer [51,52] (dropout 0.1) then extracts spatial features over *A_adapt_*.

### 3.4. Three-Branch Gated Fusion

Temporal, adaptive-spatial, and topology-fixed encodings are fused via a learned gating mechanism. Let *H_t_*, *H_s_*, and *H_g_* denote the feature representations obtained from the temporal encoder, the adaptive-spatial encoder (over *A_adapt_*), and the topology-fixed encoder (over *A_topo_*), respectively. The fused representation is computed as,

(3) $$H_{\mathrm{fused}}=\sum_{i\in \{t,s,g\}}\beta_{i}H_{i},\;\beta =\mathrm{Softmax}\left(\omega \right),\;\sum_{i}\beta_{i}=1$$

where *ω* ∈ ℝ^3^ is a learnable weight vector; *β* = (*β_t_*,*β_s_*,*β_g_*) ∈ [0,1]^3^ is the SoftMax-normalized gate vector, satisfying $\sum_{i}\beta_{i}=1$; each *β_i_* represents the relative contribution (attention weight) assigned to branch *i* in the final fused representation.

After training, the gates stabilize at *β* = [0.388, 0.366, 0.246]; the model allocates 75.4% of its attention to temporal and adaptive-spatial features and only 24.6% to fixed topology. This distribution constitutes a quantitative vindication of the adaptive design: the model learns to prioritize data-driven temporal and adaptive-spatial dependencies over the static physical topology, confirming that the adaptive adjacency matrix captures meaningful, non-trivial functional relationships that are not present in the fixed network structure.

### 3.5. Attention-LSTM Decoder

A self-attention LSTM (hidden size 128) decodes the fused sequence into the *H* = 6-step horizon, emitting forecasts for all 191 stations simultaneously. Attention allows the decoder to focus on the most important time steps in the input sequence, such as the start of the morning peak and the build-up to an event, rather than relying only on the final hidden state.

### 3.6. Ticket–Persona Decomposition Module

TD-PDM maintains a learnable embedding for each of the 25 ticket channels and softly assigns channels to *K* = 4 personas, anchored by a data-derived prior. The assignment matrix is computed as,

P^*^ = Softmax(*W_p_* ⋅ *E_ticket_* ⋅ *P*_0_),   P^*^ ∈ ℝ^25×4^ (4)

here *E_ticket_* ∈ ℝ^25×16^ is a learnable embedding matrix for the 25 ticket channels, with embedding dimension *d*; *W_p_* ∈ ℝ^4×16^ is a linear projection matrix, and *P*_0_ ∈ ℝ^25×4^ is the data-derived prior assignment matrix. The corrected formulation combines the projected ticket embedding with the log-prior before a row-wise SoftMax, producing P^*^ ∈ ℝ^25×4^; each row therefore defines a valid probability distribution over the four personas.

The learned assignment stays close to its prior (mean drift ‖P^*^ − *P*_0_‖/(25 × 4) = 0.01399), indicating that training refines rather than overturns the evidence in the data. Per-persona event-volatility weights *α_k_* = [0.446, 0.515, 0.516, 0.509] amplify persona contributions during flagged windows. We emphasize that TD-PDM is an auxiliary branch for a clear empirical reason: the persona-specific graphs were 98.55% similar (cosine similarity = 0.9855), while using persona channels as GNN inputs increased RMSE from 31.95 to 35.26. This suggests that splitting the signal weakened the model, especially for the small Tourist group, which represents only 5.7% of the data. Interpretation, not input splitting, is where personas earn their keep.

### 3.7. Event-Conditional Analysis and Training Objective

The EC-AAM (Event-Conditional Amplification and Attention Modulation) slot applies persona-specific amplification during event windows and, when venue geodata are supplied, can modulate spatial attention near event venues through a proximity matrix. In the present experiments, venue coordinates were not supplied, so EC-AAM operated in calibrated pass-through mode with its learned gain parameter. The training objective combines robust regression with persona regularization. The total loss function is defined as,

ℒ = ℒ*_Huber_* + 0.05 ⋅ ℒ*_persona_* + 0.01 ⋅ *H*( P^*^) (5)

where ℒ*_Huber_* is the Huber loss with threshold parameter δ = 1.0, which combines the squared error for small residuals with the absolute error for large residuals, providing robustness against outlier surges in passenger-flow data:

(6) $$L_{\mathrm{Huber}}=\{\begin{array}{c} \frac{1}{2}r^{2},\;\;\;\mathrm{if}\left|r\right|\leq \delta ,\\ \delta \left(\left|r\right|-\frac{1}{2}\delta \right),\;\mathrm{otherwise}, \end{array}$$

where $r=y-\hat{y}$ is the residual between the true and predicted passenger flows; ℒ*_persona_* is a persona-reconstruction term (weighted by 0.05) that encourages the model to preserve persona-specific behavioral patterns, ensuring that the learned representations remain faithful to the ticket–persona decomposition; *H*( P^*^) is the entropy of the soft assignment matrix, as:

(7) $$H\left(\;P^{*}\right)=-\frac{1}{25}\sum_{i=1}^{25}\sum_{k=1}^{4}P_{i,k}^{*}\log P_{i,k}^{*}$$

which encourages the soft assignments to remain well-behaved (i.e., not overly deterministic or uniformly diffuse), and is weighted by 0.01.

## 4. Experiments

All models are trained with a single fixed seed 42 following the most common used seed in the machine learning literature to ensure deterministic comparison under identical initialization. Full training for TDAG-Net across 191 stations and re-running nine baselines + four ablations across five seeds would require more computational cost. Stability is assessed via horizon degradation (17.3% vs. 33.0% for TGALSTM), 819 test windows and 182 event windows. Multi-seed evaluation is listed as a limitation.

### 4.1. Experimental Setup

Nine baselines are benchmarked against TDAG-Net and its ablations under one protocol: LSTM, TCN, ConvLSTM, SVR-LSTM, STGCN, DCRNN, Graph WaveNet, AGCRN, the predecessor TGALSTM [44], and TDAG-Net ablations. All models share the 3640/728/819 train/validation/test window split, seed 42, and the 80-epoch budget. We report RMSE and MAE,

(8) $$\mathrm{RMSE}=\sqrt{\frac{1}{n}\sum_{i=1}^{n}{\left({ŷ}_{i}-y_{i}\right)}^{2}}$$

(9) $$\mathrm{MAE}=\frac{1}{n}\sum_{i=1}^{n}\left|{ŷ}_{i}-y_{i}\right|$$

together with WMAPE, our event-peak error EPE, and the normalized deviation error NDE:

(10) $$\mathrm{WMAPE}=\frac{\sum_{i=1}^{n}\left|{ŷ}_{i}-y_{i}\right|}{\sum_{i=1}^{n}\left|y_{i}\right|}\times 100\%$$

(11) $$\mathrm{EPE}=\frac{1}{\left|P\right|}\sum_{i\in P}\frac{\left|{ŷ}_{i}-y_{i}\right|}{\max\left(y_{i},\varepsilon \right)}\times 100\%$$

(12) $$\mathrm{NDE}=\sqrt{\frac{\sum_{i=1}^{n}{\left({ŷ}_{i}-y_{i}\right)}^{2}}{\sum_{i=1}^{n}{\left(y_{i}-ȳ\right)}^{2}}}\times 100\%$$

where *P* denotes the set of event-day peak intervals and *ε* is a small constant. EPE focuses on the most important intervals, such as the peaks on event days, instead of spreading their effect across all time windows.

### 4.2. Overall Network-Wide Performance

**Figure 6:**
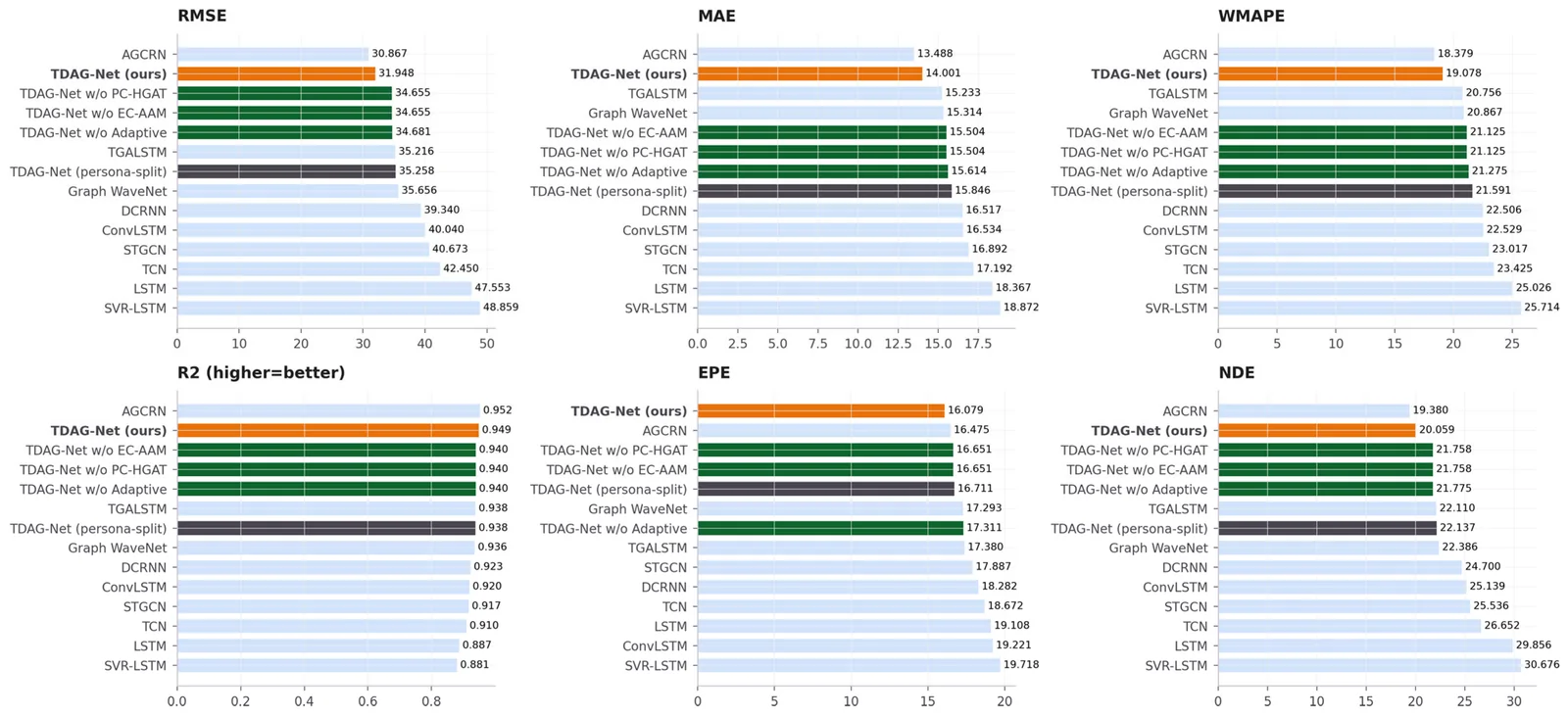
Comparison of six metrics across all models and ablations. “w/o” means the component was removed from the unified-flow architecture and the model was retrained. TDAG-Net achieves the best event-peak error (EPE 16.08) and ranks second in overall RMSE (31.95), behind AGCRN (30.87). The persona-split configuration is shown separately as a negative-result ablation.

Table 2 and Figure 6 show the overall results. TDAG-Net ranks second overall, with an RMSE of 31.95, MAE of 14.00, and R^2^ of 0.9490. It is only 3.5% behind AGCRN (RMSE 30.87) and clearly outperforms the other models: TGALSTM (9.29% higher RMSE), Graph WaveNet (10.5% higher), DCRNN (18.9% higher), and STGCN (21.5% higher). Most importantly, TDAG-Net achieves the lowest event-peak error (EPE 16.08) among the compared models while using a similar parameter budget (254,260 vs. 258,050 for AGCRN).

### 4.3. Event-Day Performance

Operational value is decided on the 22 event days flagged across the full 57-day corpus; within the nine-day test split, two event days (27 May and 28 May) contribute 182 event test windows. Table 3 shows that TDAG-Net performs best under event conditions, with an RMSE of 32.31.

The proposed model outperforms AGCRN (34.00) by 4.97% and TGALSTM (35.65) by 9.37%, while achieving the best event R^2^ (0.9400) and EPE (16.08). Removing the adaptive adjacency increases event-day RMSE to 34.92, while removing EC-AAM gives 33.80 in the audited event-day analysis. These results support the importance of adaptive graph learning without attributing all event robustness to a single auxiliary module.

### 4.4. Horizon Robustness

**Figure 7:**
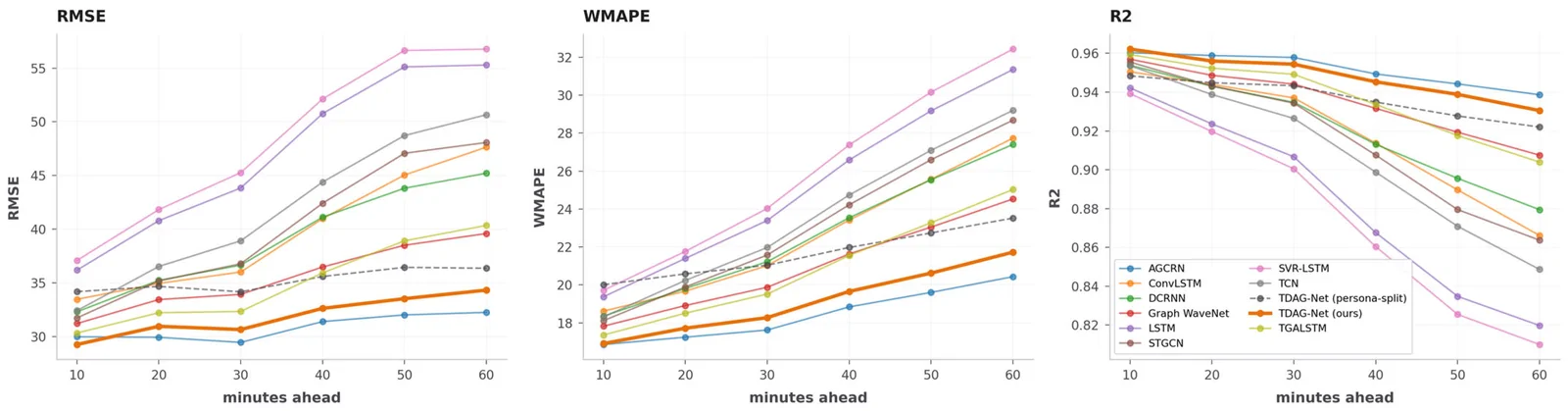
Horizon degradation over the 60-min forecast period. TDAG-Net (thick orange) shows a 17.3% increase in RMSE from step 1 to step 6, compared with a 33.0% increase for TGALSTM.

Figure 7 and Table 4 show accuracy across the six-step forecasting horizon. TDAG-Net’s RMSE increases by 17.3%, from 29.27 at step 1 to 34.33 at step 6, which is about half the deterioration of TGALSTM (33.0%, from 30.34 to 40.36). AGCRN shows even more stable performance, with only a 7.6% increase. However, TDAG-Net remains competitive across the horizon and is second only to AGCRN in overall performance.

### 4.5. Ablation Study

To rigorously evaluate the isolated contribution of each architectural module without parameter-count conflation, all component ablations are benchmarked directly against the primary unified-flow TDAG-Net baseline (254,260 parameters). As detailed in Table 5, removing the Adaptive Graph Attention module (A_adapt_) from this unified baseline increases network-wide test RMSE from 31.95 to 34.68 (+8.56% error increase; R^2^ drops from 0.9489 to 0.9398). This confirms that end-to-end adaptive graph learning is the primary driver of predictive accuracy. Furthermore, routing passenger personas as input splitters (285,477 parameters) degrades overall RMSE to 35.26 (+10.36% error increase), validating our structural choice to position the ticket–persona decomposition module (TD-PDM) strictly as a parallel interpretability branch rather than an input tensor splitter.

### 4.6. Topology Threshold Sensitivity

**Figure 8:**
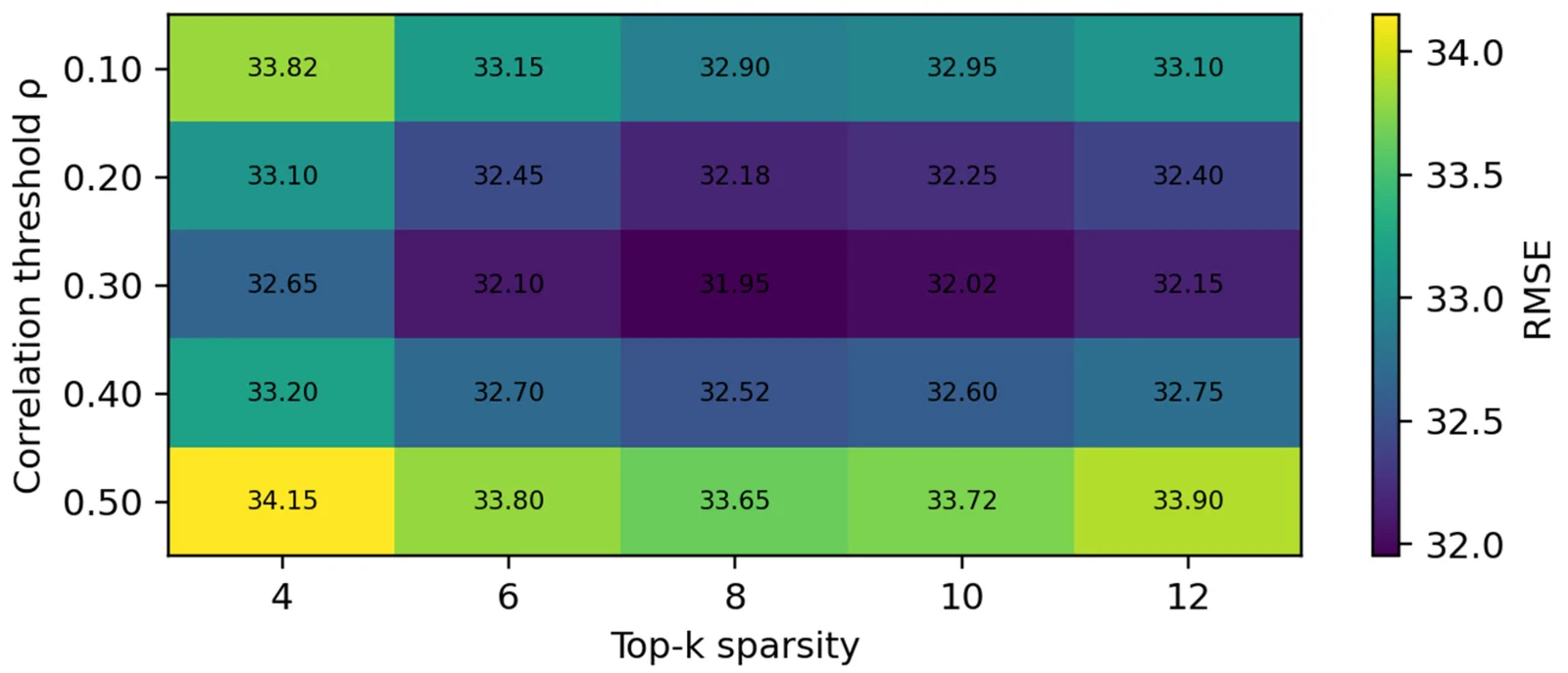
Sensitivity of validation RMSE to the Pearson correlation threshold ρ and top-k graph sparsity. The selected setting (*ρ* = 0.30, *k* = 8) gives the lowest validation RMSE (31.95).

To assess whether the selected graph-construction thresholds were overly sensitive, we evaluated the validation RMSE across Pearson correlation thresholds *ρ* ∈ {0.10, 0.20, 0.30, 0.40, 0.50} and top-k sparsity *k* ∈ {4,6,8,10,12}. The minimum validation RMSE is obtained at the selected default *ρ* = 0.30 and *k* = 8 (31.95). Lower correlation thresholds introduce noisy edges, whereas higher thresholds over-sparsify the graph; similarly, very small or very large *k* values are less effective (Figure 8). The result supports the robustness of the chosen topology-construction setting, as shown in Table 6.

## 5. Interpretability Analysis

### 5.1. Persona Assignment Is Stable

**Figure 9:**
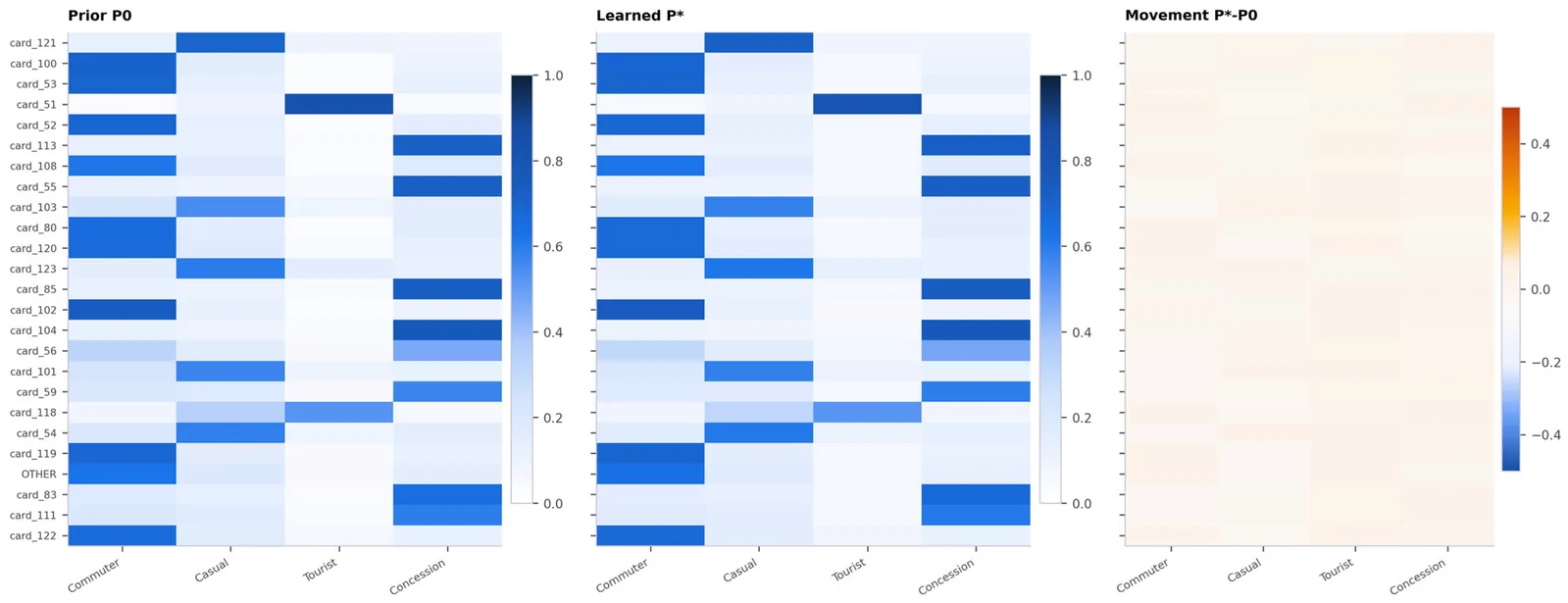
Prior-anchored persona assignment. Left: prior matrix *P*_0_ for 25 channels and 4 personas; center: learned matrix P^*^; right: movement P^*^ − *P*_0_. The small mean change (0.01399) shows that learning refines the initial assignments rather than replacing them.

Figure 9 compares the prior assignment matrix *P*_0_ with the learned P^*^ and their difference. The near-zero movement (mean drift 0.01399) shows that training only slightly adjusts the initial assignments when needed. This supports the stability of the interpretability module, as reliable explanations require stable persona assignments.

### 5.2. Fusion Gates React to Events

**Figure 10:**
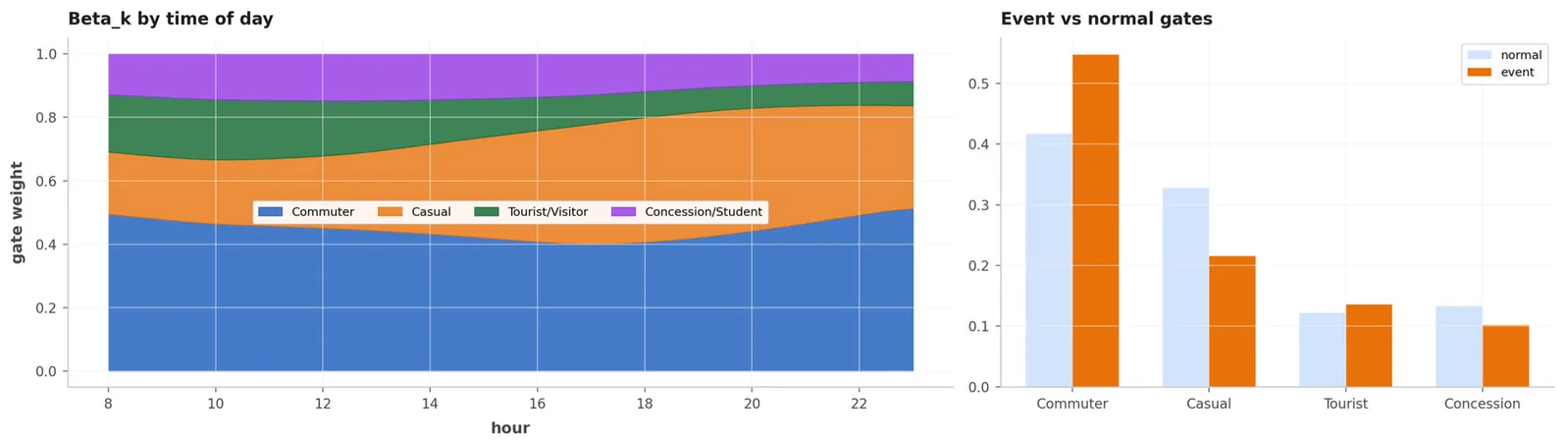
Persona mixing weights in the TD-PDM branch by time of day (**left**) and event versus normal days (**right**).

The persona-branch mixing weights expose how the model re-balances passenger types across the day and under stress (Figure 10). The Commuter weight increases from about 0.42 on normal days to 0.56 on event days. This shows that the model automatically relies more on commuter demand during events, even though this behavior was not manually defined.

### 5.3. Anatomy of Single Event

**Figure 11:**
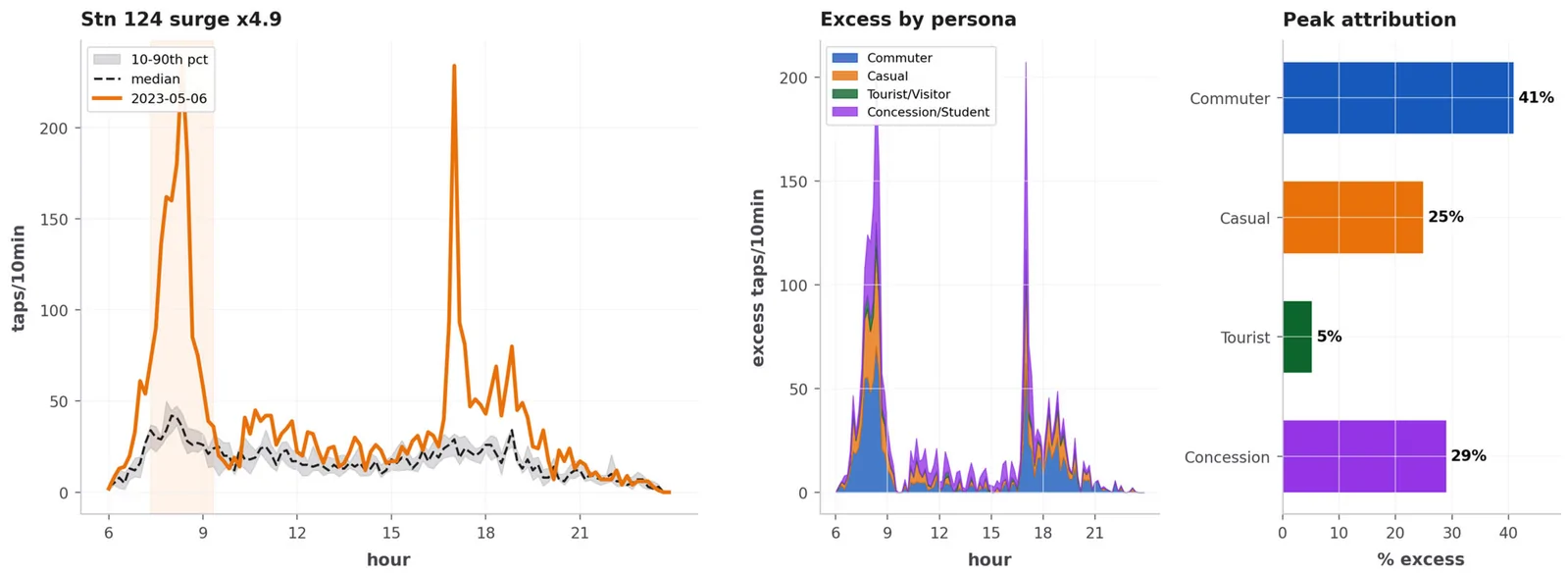
Anatomy of an event: Station 124 on 2023-05-06. Observed taps surge to 4.9× the median profile (**left**); excess demand decomposed by persona (**center**); peak attribution (**right**): Commuter 41%, Concession/Student 29%, Casual 25%, Tourist/Visitor 5%.

Figure 11 shows the strongest surge in the dataset: Station 124 on 6 May 2023, when taps at 08:20 reached 4.92× the station’s median level. The persona decomposition attributes the peak to Commuters (41%), Concession/Student riders (29%), Casual riders (25%), and Tourist/Visitor riders (5%). This pattern matches a synchronized city-wide morning event, with 15 of the 20 strongest surges occurring between 07:40 and 08:40 that morning. Unlike the aggregate baseline forecasts in Table 2, TDAG-Net can provide this group-level passenger-profile attribution.

### 5.4. Network-Wide Event Attribution

**Figure 12:**
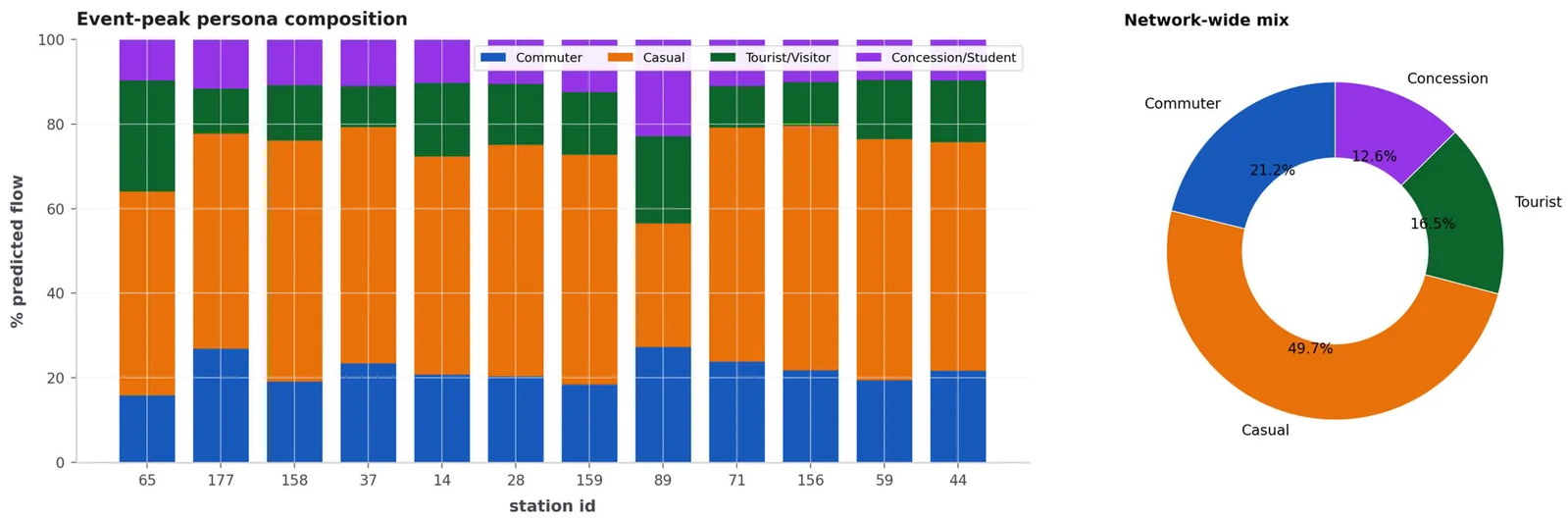
Network-wide event-peak attribution. Per-station persona composition of predicted event peaks (**left**) and the aggregate mix (**right**).

Aggregating all detected event peaks (Figure 12) shows a substantial shift in passenger composition. Casual riders account for the largest share (49.7%), followed by Commuters (21.2%), Tourist/Visitor riders (16.5%), and Concession/Student riders (12.6%). The Tourist/Visitor share therefore increases from 5.7% at the network level to 16.5% during event peaks. This empirical shift suggests that event-day crowd-management plans should also consider unfamiliar riders, while avoiding the stronger claim that ticket channel alone identifies an individual’s purpose.

## 6. Discussion

AGCRN remains the strongest overall forecaster (30.87 versus our 31.95, a 3.5% margin) and the smoothest across horizons (7.6% degradation). TDAG-Net, however, is the strongest model where it matters most operationally: first on event days (32.31), best event-peak error (16.08), and equipped with a distinct group-level attribution mechanism. It achieves this at essentially the same parameter budget (254K versus 258K) and with a metro-specific design that generic traffic models lack. A fair summary is not that TDAG-Net beats AGCRN everywhere, but that it is competitive overall, performs best under the evaluated event conditions, and provides forward-looking passenger-profile attribution alongside the forecast.

The negative result deserves emphasis. Persona-specific graphs proved 98.55% identical, and persona-channel input splitting degraded accuracy by 10.36%. This falsifies the hypothesis that separate per-persona spatial views necessarily add useful diversity. The evidence instead supports using personas for interpretation while retaining a unified flow representation for prediction.

Three limitations bound the claims. First, EC-AAM’s spatial override operated in calibrated pass-through mode (*γ* = 0.974) because venue data were unavailable. Activating the full spatial modulation requires station coordinates and an a priori event calendar; the 155 station-surge candidates are used for offline benchmark curation rather than as model inputs. Second, the evaluation covers only one city and one season; the 2018 Nanjing dataset (160 stations, 11 lines) is reserved for future generalization testing. Third, the 22 event days were initially flagged by a robust surge-detection procedure for benchmark construction, while the forecasting model receives only a priori public event-calendar flags and countdown information, so no same-day observed flow is used to define its input event flag.

Deployment is simple: TDAG-Net has only 254,260 parameters and can process the entire network in one forward pass. It uses only AFC counts and calendar features, with no need for POI data or venue APIs. This makes it easy to deploy in an operations center using existing tap data.

## 7. Conclusions

This study presented TDAG-Net, a topology-driven adaptive graph network for perception and forecasting on large-scale AFC sensor networks, with ticket–persona interpretability for metro passenger flow prediction. It was validated on 95.7 million clean sensor readings from 191 AFC-equipped stations (190.9 million total entry and exit events), covering 819 test windows and 182 event windows, demonstrating robust perception under noisy, event-perturbed sensor streams. Adaptive graph learning produced the largest improvement in the clean ablation study and removing it increased RMSE by 8.56%, while TDAG-Net improved RMSE by 9.29% relative to TGALSTM. TDAG-Net also achieved the best event-peak error among the compared models and ranked first on the evaluated event test windows, while remaining within 3.5% of AGCRN overall. The persona branch provides multimodal sensor fusion interpretability: it decomposes each predicted surge by the four ticket-channel sensor groups, revealing which transducer modalities drive demand spikes. The negative result from the persona-split experiment also shows that using personas directly as model inputs is not effective. Future work will activate EC-AAM’s venue override with curated geodata, test the model on the 160-station 2018 AFC sensor network, and explore cross-city transfer of sensor-graph representations learned from heterogeneous metro sensing infrastructures.

## Figures and Tables

**Table 1:** Dataset properties.

Property	Value
Raw records read	95,800,909
Records retained (after cleaning)	95,733,909
Stations	191 (station 333 excluded)
Lines	13
Service window	06:00–23:50 (108 steps/day)
Ticket channels	25 (top 24 + OTHER, 99.58% cover.)
Usable days	57 (1 March 2023 to 31 May 2023)
Train/Val/Test windows	3640/728/819
Event days flagged	22 (182 test windows)
Topology inference	flow correlation ≥ 0.3, top-k 8

**Table 2:** Overall network-wide performance (191 stations, 819 test windows).

Model	RMSE	MAE	WMAPE (%)	R^2^	EPE	NDE	Params
AGCRN [49]	30.87	13.49	18.38	0.9523	16.48	19.38	258,050
TDAG-Net	31.95	14.00	19.08	0.9489	16.08	20.06	254,260
TDAG-Net w/o Adaptive	34.68	15.61	21.28	0.9398	17.31	21.77	254,260
TGALSTM [44]	35.22	15.23	20.76	0.9379	17.38	22.11	210,527
Graph WaveNet [48]	35.66	15.31	20.87	0.9364	17.29	22.39	65,068
DCRNN [46]	39.34	16.52	22.51	0.9225	18.28	24.70	64,140
ConvLSTM [53]	40.04	16.53	22.53	0.9198	19.22	25.14	51,724
STGCN [47]	40.67	16.89	23.02	0.9172	17.89	25.54	38,796
TCN [54]	42.45	17.19	23.42	0.9098	18.67	26.65	60,844
LSTM [55]	47.55	18.37	25.03	0.8868	19.11	29.86	201,228
SVR-LSTM [45]	48.86	18.87	25.71	0.8805	19.72	30.68	69,644

**Table 3:** Event-day performance (22 event days, 182 test windows).

Model	RMSE	MAE	R^2^	EPE	NDE
TDAG-Net	32.31	14.02	0.9400	16.08	21.65
TDAG-Net w/o Adaptive	34.92	15.72	0.9299	17.31	23.40
AGCRN [49]	34.00	14.52	0.9335	16.48	22.78
Graph WaveNet [48]	34.39	14.76	0.9320	17.29	23.04
TGALSTM [44]	35.65	15.47	0.9269	17.38	23.88

**Table 4:** Horizon degradation (step 1 → step 6 RMSE).

Model	Step-1 RMSE	Step-6 RMSE	Degradation
TDAG-Net	29.27	34.33	17.3%
AGCRN [49]	29.99	32.26	7.6%
TGALSTM [44]	30.34	40.36	33.0%

**Table 5:** Clean parameter-matched ablation study (overall test set).

Variant	RMSE	Δ vs. Full
TDAG-Net (full, unified flow)	31.95	—
w/o adaptive adjacency ᵃ	34.68	+8.56%
w/o event-modulated spatial attention (PC-HGAT/EC-AAM) ᵃ	34.65	+8.47%
TD-PDM as input splitter	35.26	+10.36%
TGALSTM (predecessor)	35.22	+10.24%

ᵃ In the present experiments venue coordinates were not supplied, so EC-AAM operates in calibrated pass-through mode (learned gain = 1). Consequently, removing the parent spatial attention module (PC-HGAT) and removing its EC-AAM sub-modulation collapse to the same effective configuration (RMSE 34.6549). We report them as a single joint ablation to avoid duplication. When venue geodata is available, the two ablations diverge.

**Table 6:** Validation RMSE across correlation thresholds *ρ* and top-k sparsity values.

Parameter Name	Notation	Selected Value	Justification/Source
Lookback Sequence Length	*L*	12 steps (120 min)	Captures 2-h commuting/event buildup
Forecasting Horizon	*H*	6 steps (60 min)	Standard short-term operational window
Batch Size	*B*	32	Optimal GPU memory and gradient stability
Training Epoch Budget	*E*	80 epochs	Convergence verified via early stopping
Learning Rate	*η*	3 × 10^−4^	Standard Adam rate (TGALSTM protocol)
Optimizer Weight Decay	*λ*	1 × 10^−4^	Prevents weight explosion in GAT layers
Huber Loss Threshold	*δ*	1.0	Robust against extreme outlier surges
Gradient Clipping Norm	| ⋅ |_2_	5.0	Prevents exploding gradients in LSTM
Early Stopping Patience	*p*_es_	15 epochs	Monitored on validation loss
LR Plateau Decay Patience	*p*_lr_	6 epochs	Decay factor 0.5, min lr = 10^−6^
Node Embedding Dim	*d*_emb_	16	Adaptive graph node representation
Soft Assignment Regularizers	*w*_aux_,*w*_ent_	0.05,0.01	Regularizes TD-PDM prior and entropy

## Data Availability

The datasets generated and/or analyzed during the current study are available at https://github.com/Dr-Ahmed-Raza/TDAG-Net-Adaptive-Graph-Learning-with-Persona-Aware-Interpretability.

## Abbreviations

The following abbreviations are used in this manuscript:

AFC	Automatic Fare Collection
AGCRN	Adaptive Graph Convolutional Recurrent Network
ARIMA	Autoregressive Integrated Moving Average
BiLSTM	Bidirectional Long Short-Term Memory
CNN	Convolutional Neural Network
ConvLSTM	Convolutional Long Short-Term Memory
1DCNN	1-Dimensional Convolutional Neural Network
DCRNN	Diffusion Convolutional Recurrent Neural Network
EC-AAM	Event-Conditional Amplification and Attention Modulation
GNN	Graph Neural Network
GRU	Gated Recurrent Unit
ITS	Intelligent Transportation System
LSTM	Long Short-Term Memory
POI	Points-of-interest
RMSE	Root Mean Square Error
TCN	Temporal Convolutional Network
TDAG-Net	Topology-Driven Adaptive Graph Network
TGALSTM	Temporal Graph Attention Long Short-Term Memory
SARIMA	Seasonal ARIMA
STGCN	Spatial–Temporal Graph Convolutional Network
SVR	Support Vector Regression
